# Chronic Periodontal Disease May Influence the Pulp Sensitivity Response: Clinical Evaluation in Consecutive Patients

**DOI:** 10.5402/2012/246875

**Published:** 2012-04-05

**Authors:** Elizangela Partata Zuza, Ana Luiza Vanzato Carrareto, Ana Emília Farias Pontes, Marcelo Brunozzi, Juliana Rico Pires, Benedicto Egbert Corrêa Toledo

**Affiliations:** ^1^Department of Master of Dental Science, School of Dentistry, Educational Foundation of Barretos (UNIFEB), Avenide Roberto Frade Monte 389, 14783-226, Barretos, SP, Brazil; ^2^School of Dentistry, Educational Foundation of Barretos (UNIFEB), 14783-226, Barretos, SP, Brazil

## Abstract

*Purpose*. The aim of the present study was to evaluate the clinical response of the pulp in teeth with chronic periodontitis. *Methods*. Consecutive patients who had been admitted to the Clinics of Periodontology and fulfilled the criteria of inclusion were enrolled from January to December 2007. Ninety-eight single-root teeth from 27 patients with chronic periodontitis were evaluated clinically with regard to clinical attachment level (CAL), probing depth (PD), and gingival recession (REC). After periodontal measurements, Pulpal Sensitivity (PS) was evaluated with the use of a cooling stimulus test. Data was analyzed with Student's *t* test and contingency C coefficient. *Results*. Teeth that responded positively to PS test presented lower values of CAL (7.8 ± 2.8 mm), PD (5.0 ± 2.3 mm), and REC (2.8 ± 1.8 mm) in comparison to those that responded negatively (CAL = 12.0 ± 2.2 mm; PD = 7.9 ± 1.6 mm; REC = 4.1 ± 2.4 mm) (*P* < 0.01, Student's *t* test). In addition, significant correlations were observed between PS and periodontal parameters. *Conclusions*. Within the limits of this study, it could be suggested that the progression of periodontitis may significantly influence the negative pulpal response.

## 1. Introduction

The American Academy of Periodontology included a group of combined endodontic-periodontal lesions in the Classification for Periodontal Disease and Conditions, when there is a simultaneous occurrence of endodontic and periodontal lesions at the same tooth. The simultaneous occurrence of both conditions suggests that an interrelation may occur between the pulp tissue and periodontium [1].

The differential diagnosis of periodontal and endodontic lesions can be normally established [2], since lesions from endodontic origin show clinical and radiographic signs located nearer to the apical region, whereas the periodontal alterations are otherwise located next to marginal periodontium [3]. Diagnosis tends to be more reliable if the patient has been followed up for some period of time [4].

 However, the differential diagnosis is more obscure for the combined periodontal-endodontic lesions, since it is difficult to identify the primary etiology [5]. Thus, diagnosis must be based on the matching of the history reported by the patient, clinical examination, radiographic observations, and complementary tests [6]. These tests are usually related to the determination of pulpal sensitivity [7], radiographic contrasts [4], type of communication with the gingival margin [8], and microbiological analyses [9].

Several studies have shown that the communication between pulp and periodontium may occur not only via apical foramen, but also via accessory canals [10, 11] or dentinal tubules [12]; hence, endodontic and/or periodontal alterations do not have to reach the apical level for the interrelation establishment. The moment in which the periodontal diseases begin to influence the clinical response of the pulp is still unknown, and it is relevant to establish an early differential diagnosis and an appropriate treatment. Thus, the aim of the present study was to evaluate the clinical response of the pulp in teeth presenting chronic periodontitis.

## 2. Materials and Methods

This study was approved by the Ethics Committee in Research of the Educational Foundation of Barretos (UNIFEB) (no. 47/2006). All patients that presented to the Clinics of Periodontology in the year of 2007 and that fulfilled the criteria of inclusion signed up the informed consent form to be included in the present study. Ninety-eight teeth from 27 patients (mean age 32.3 ± 6.3 years) were included in the study.

 The following inclusion criteria were considered: patients aging between 18 and 40 years, regardless of sex or race, who had not been submitted to periodontal treatment up to 6 months before the study, and presenting with diagnosis of chronic periodontitis. This diagnosis was based on the criteria described by Flemmig [13].

Only teeth with no signs of caries, restoration, attrition, abrasion, erosion, or occlusal trauma were enrolled in this study. Each experimental tooth presented a diagnosis of chronic periodontitis, while a contralateral tooth was used as a control with no clinical attachment loss (apical migration of the junctional epithelium). The following parameters were evaluated: probing depth (PD), gingival recession (REC), and clinical attachment level (CAL). The REC was considered as the distance from the cement-enamel junction (CEJ) to the gingival margin, while CAL was defined as the distance from the CEJ to the bottom of the pocket. Six sites per tooth were measured (mesiobuccal, buccal, distobuccal, mesiolingual, lingual, and distolingual) with a Williams's periodontal probe (Hu-Friedy, Chicago, IL, USA).

After periodontal measurements, the pulpal sensitivity (PS) test was performed by cooling stimulus with a refrigerant spray at −50°C (Endo Frost, Cold Spray, Roeko, Langenau, Germany). The relative isolation was performed with cotton rolls, and a small cotton pellet was embedded with the cooling air jet activated by the valve pressure for approximately 3 seconds. The cotton pellet was applied on the buccal-cervical surface of the tooth, and the results were recorded as a positive response (expressed as “1”) or as a negative response (expressed as “0”).

### 2.1. Statistical Analysis

Two examiners were trained and calibrated for this study; the first one (EPZ) performed the clinical measurement (standard error = 0.55 mm, for CAL), while the second one (ALVC) performed sensitivity pulp test. Intraexaminer reliability was determined after evaluating 18 teeth (6 single-root teeth randomly selected in 3 patients) in two different occasions, a week apart.

The CAL, PD, and REC were analyzed using a specific program (BioEstat 5.0, Sociedade Civil Mamirauá/MCT—CNPq, PA, Brazil), considering the tooth as unity of analysis. The level of significance was considered as 5%. The site with higher clinical attachment loss was selected as representative of the tooth. Student's *t* test was applied to test differences between teeth that responded positively versus teeth that responded negatively to PS. Contingency coefficient C was calculated to evaluate correlation between periodontal parameters (CAL, PD and REC) and PS.

## 3. Results

Sixty-eight teeth responded positively to the sensitivity test, while 30 of them did not respond. Tables 1, 2 and 3 showed the distribution of CAL, PD, and REC in relation to PS. All the control teeth responded positively.

**Figure 1:**
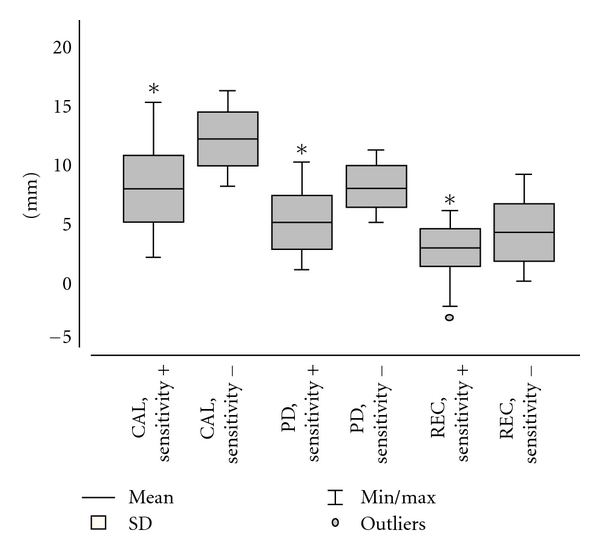
Comparisons between sites which responded differently to pulpal sensitivity. *Difference statistically significant in comparison to teeth with negative sensitivity (*P* < 0.01, Student's *t* test).

 Comparisons between sites which responded differently to PS test are presented in a Box-Plot graphic in Figure 1. Teeth with positive sensitivity presented lower values of CAL (7.8 ± 2.8 mm) than those with negative sensitivity (CAL = 12.0 ± 2.2 mm) (*P* < 0.0001, Student's *t* test). Values from PD were significantly lower in teeth that responded positively (PD = 5.0 ± 2.3 mm) than in those that responded negatively to the pulpal sensitivity test (PD = 7.9 ± 1.6 mm) (*P* < 0.0001, Student's *t* test).

The REC values were also lower (REC = 2.8 ± 1.8 mm) in teeth with positive response than in those that responded negatively (REC = 4.1 ± 2.4 mm) (*P* < 0.002, Student's *t* test). Significant correlations were verified between Pulpal sensitivity and the parameters of CAL (Contingency C Coefficient = 0.5019, *P* < 0.0001), PD (Contingency C Coefficient = 0.5454, *P* < 0.0001) and REC (Contingency C Coefficient = 0.5500, *P* < 0.0001) (Table 4).

## 4. Discussion

Although several studies have clearly shown the interrelation between pulp and periodontium, there are still controversies [11]. Authors such as Mazur and Massler [14] reported that periodontal disease does not influence the pulp, suggesting that pulpal degeneration could occur due to systemic factors; however, our findings showed that the progression of periodontitis may significantly influence pulpal response to the stimulus, with higher levels of CAL, PD, and REC in sites with negative response. This is in line with histological studies, which have also showed pulp alterations in teeth with periodontal diseases [15, 16].

The influence of the pulp tissue upon periodontal structures is more evident than the influence of periodontium on the pulp. If pulp tissue is exposed to a frequent low-intensity long-lasting stimulus, it would feature a slow and asymptomatic chronic-degenerative reaction with consequent pulpal necrosis [17]. The results of the present study sustain this hypothesis, since CAL, PD, and REC were correlated to a negative pulpal response. These findings showed that the pulp condition depended on the level of severity of the active periodontal disease, in agreement with Cardon et al. [18] that found a statistically significant correlation between the periodontal attachment loss and the negative pulpal sensitivity.

Some competing causes were excluded in our study, such as occlusal traumas, decayed teeth, teeth featuring restorations, and prosthetic crowns. For the reliability, it was just included single-root teeth, eliminating the possibility of false positives, which is often found in multiroot elements. Moreover, the pulpal sensitivity verification was performed using a cooling stimulus test, which enables a reliable diagnosis of a necrotic pulp in 90% of the cases (with few false negatives) [19]. The use of dry ice and refrigerant spray provides the most consistent stimuli, whereas heated gutta-percha and hot water are highly variable [20]. In addition, it is much rarer to have false positive to cold than to electrical test [21]. On the other hand, the application of a single test was not sufficient to a conclusive diagnostic of the pulp sensitivity [22], which shows a limitation. Besides, the positive response to the cooling stimulus test shows clinical pulp vitality, but it does not means histological signs of normality, since histological evaluations were not made.

Our findings showed that teeth with CAL value as higher as 8 mm and PD as deep as 5 mm, without reaching the tooth apex, may present negative response to pulpal stimulus. Then, it is not necessary that the periodontal alterations reach the apical foramen region to establish a relationship between the periodontal and pulpal tissues [4, 19, 23].

There is a scarceness of scientific evidence that clarifies the different aspects of the relation between periodontal and endodontic alterations [19]. Some lines of evidence must still be clarified, such as whether periodontal disease may cause pulpal necrosis and whether a devitalized tooth may cause periodontitis. Further clinical and histological investigations are needed to better explain the interrelation between both conditions.

## 5. Conclusions

Within the limits of this study, it could be suggested that the progression of periodontitis may significantly influence the negative pulpal sensitivity.

## Figures and Tables

**Table 1:** Distribution of sites according to clinical attachment level (CAL), in teeth with positive and negative pulpal sensitivity response.

CAL value	Positive pulpal sensitivity (*n* = 68)	Negative pulpal sensitivity (*n* = 30)
2 mm	1	0
3 mm	3	0
4 mm	9	0
5 mm	2	0
6 mm	2	0
7 mm	11	0
8 mm	13	1
9 mm	11	3
10 mm	6	4
11 mm	5	4
12 mm	3	8
13 mm	0	2
14 mm	0	4
15 mm	2	1
16 mm	0	3

**Table 2:** Distribution of sites according to probing depth (PD), in teeth with positive and negative pulpal sensitivity response.

PD value	Positive sensitivity (*n* = 68)	Negative sensitivity (*n* = 30)
1 mm	6	0
2 mm	9	0
3 mm	4	0
4 mm	1	0
5 mm	19	3
6 mm	10	3
7 mm	13	5
8 mm	3	9
9 mm	0	5
10 mm	3	4
11 mm	0	1

**Table 3:** Distribution of sites according to gingival recession (REC), in teeth with positive and negative pulpal sensitivity response.

REC value	Positive pulpal sensitivity (*n* = 68)	Negative pulpal sensitivity (*n* = 30)
−3 mm	1	0
−2 mm	1	0
0 mm	4	1
1 mm	4	1
2 mm	23	8
3 mm	11	3
4 mm	12	6
5 mm	9	4
6 mm	3	0
7 mm	0	3
8 mm	0	3
9 mm	0	1

**Table 4:** Contingency C coefficient between pulpal sensivity response and CAL, PD, and REC (*n* = 98).

	Contingency C Coefficient	*P* value
CAL × Pulpal Sensitivity	0.5019	<0.0001*
PD × Pulpal Sensitivity	0.5454	<0.0001*
REC × Pulpal Sensitivity	0.5500	<0.0001*

CAL: clinical attachment level; PD: probing depth; REC: gingival recession; *Value statistically significant.
